# Hematocrit Values Predict Carotid Intimal-Media Thickness in Obese Patients With Non-Alcoholic Fatty Liver Disease: A Cross-Sectional Study

**DOI:** 10.3389/fendo.2018.00203

**Published:** 2018-04-30

**Authors:** Giovanni Tarantino, Luigi Barrea, Domenico Capone, Vincenzo Citro, Teresa Mosca, Silvia Savastano

**Affiliations:** ^1^Department of Clinical Medicine and Surgery, Medical School, University of Naples Federico II, Naples, Italy; ^2^Department of Clinical Neurosciences, Anesthesiology and Drug-Use, Section of Clinical Pharmacology, University of Naples Federico II, Naples, Italy; ^3^Department of Internal Medicine, Umberto I Hospital, Nocera, Salerno, Italy

**Keywords:** hematocrit, carotid intima-media thickness, obesity-related non-alcoholic fatty liver disease, US, CV risk

## Abstract

**Background:**

Literature data suggest with some criticism that full-fledged cardiovascular (CV) events (acute or chronic) are likely predicted by blood components, which are reported to be associated with the presence/severity of non-alcoholic fatty liver disease (NAFLD). This study was aimed at determining which marker(s) derived from blood count, such as white blood cells, neutrophils, neutrophil/lymphocyte ratio, platelet count, hemoglobin, mean corpuscular volume, hematocrit values were associated with ear or subclinical atherosclerosis, in obese patients of various classes suffering from NAFLD.

**Methods:**

One hundred consecutive obese patients presenting NAFLD at ultrasound, with low prevalence of co-morbidities and no history or instrumental features of CV diseases, underwent carotid intima-media thickness (IMT) assessment by Doppler ultrasonography. All of them were studied taking into account anthropometric parameters, the metabolic profile, and inflammatory markers.

**Results:**

White blood cells and neutrophil count showed no statistical association with IMT, which was predicted by the amount of visceral adiposity, as appreciated by ultrasonography. After adjusting for visceral adiposity and smoking status, only age and hematocrit contextually predicted early atherosclerosis, evaluated as IMT. Visceral adiposity was a confounding factor in foreseeing IMT.

**Conclusion:**

Hematocrit values along with the patient’s age suggest an initial atherosclerosis, evaluated as IMT, and if this finding is confirmed in larger cohorts, could be added to other canonical CV risk factors. Inferences can be enhanced by future prospective studies that aim to identify the relationships between incident cardio-metabolic cases and this hematologic parameter.

## Introduction

Literature data show that both full-fledged cardiovascular (CV) events (acute or chronic) and mortality derived from CV disease are likely predicted by blood components, but there are also dissenting studies.

In fact, while high count of white blood cells is an independent predictor of coronary artery disease risk in patients of both genders (1), also after adjusting for the classical risk factors (2), further research has failed to discern any association between hematological indices and coronary calcification (3). Markers including coronary artery calcification, carotid intima-media thickness (IMT), and ankle-brachial index can be used to assess CV risk, even in patients with no symptoms of heart disease. Indeed, the coronary artery disease risk associated with high white blood cells count was comparable to those of other inflammatory markers (4). Interestingly, higher levels of white blood cells are independently related to the presence of non-alcoholic fatty liver disease (NAFLD) (5), a condition reckoned as a CV risk *per se*.

Not only with total count of white blood cells was an association of the long-term carotid artery disease-related mortality found but also with an its subtype, i.e., neutrophil count (6, 7). In keeping with the subtypes of white blood cells, attention has been directed to predict coronary artery disease using the neutrophil-to-lymphocyte ratio (8, 9). Furthermore, the neutrophil-to-lymphocyte ratio is higher in patients with the most severe form of NAFLD (10, 11), although this finding has not been confirmed (12). Literature offers contrasting data about hemoglobin levels. In fact, lower levels are independent predictor of late mortality in patients undergoing coronary artery bypass grafting (13) while higher levels seem to predict the histological severity of NAFLD (14).

A blood constituent that has captured the interest of many researchers is hematocrit, which is an independent risk factor for CV mortality (15). But, also in this context, there is some disagreement. Whereas patients with chronic cerebral infarctions had hematocrit values significantly related to the carotid IMT (16), a meta-analysis comparing hematocrit and coronary artery disease risk showed a limited prediction in disease-free subjects (17). Similarly, evaluating asymptomatic cerebrovascular damage, no relationship was found between hematocrit and IMT (18). As to NAFLD, there is evidence that hematocrit levels are independently associated with fibrosis in NAFLD patients (19). Another blood cell component showing to play a role in coronary artery disease (20) is the mean volume of platelets, while their total count predicts fibrosis severity, but not steatosis grade in patients with liver-biopsy confirmed NAFLD (21, 22).

Apart data that shows an association between advanced and well-established forms of CV disease and blood components, little evidence exists about the link between hematological parameters and early stages or subclinical CV diseases, characterized by early atherosclerosis (23) in obesity-related NAFLD patients.

As previously stated, carotid IMT is increasingly regarded as a surrogate marker for assessing the initial process of atherosclerosis, its ability relying on predicting future clinical CV end-points (24). Focusing on ultrasonographic (US) evidence of IMT, we aimed at detecting any correlations between this vascular parameter and any component of blood count in a population of obese patients, with ultrasonography (US) feature of NAFLD. We also analyzed canonical and non-canonical cardio-metabolic risk factors, such as increased age, gender, smoking status, blood pressure, body fat distribution, both systemic and hepatic insulin resistance, lipid profile—including triglyceride/high-density lipoprotein (HDL) cholesterol ratio and the atherogenic index of plasma (AIP), markers of acute and chronic inflammation, C-reactive protein (CRP), fibrinogen, ferritin, and spleen volume (25).

## Materials and Methods

This cross-sectional study was performed enrolling 100 obese patients characterized by low prevalence of co-morbidities, without any history or sign of CV disease but with US findings of NAFLD. The research protocols were approved by the Ethics Committee of the Federico II University Medical School of Naples (protocol number: 231-05). All participants provided their written informed consent to participate in this study.

### Exclusion Criteria

Previous or recent CV diseases, such as coronary artery disease, cerebrovascular disease, or peripheral artery disease were excluded on the basis of history and appropriate testing. Any viral, autoimmune, metabolic liver disease (Wilson disease, hemochromatosis, or antitrypsin deficiency) was ruled out. Celiac disease was excluded on the grounds of the IgA anti-tissue transglutaminase antibodies absence. Alcohol abuse tested according to the DSM-IV diagnostic criteria, carrying out screening tests such as MAST (Michigan Alcohol Screening Test) and CAGE (Cut down, Annoyed, Guilty, and Eye opener), as well as random tests for blood alcohol concentration and the use of a surrogate marker, e.g., mean corpuscular volume (MCV). The therapy of patients was maintained according their schedules.

### Metabolic Profile

Overweight and the class of obesity (I, II, III) was set by body mass index (BMI) cut-offs, i.e., 25–29.9, 30–34.9, 35–39.9, and >40 kg/m^2^, respectively. Abdominal obesity was assessed evaluating waist circumference (WC) measured at the midpoint between the lower border of the rib cage and the iliac crest. Hip circumference was measured around the widest part of the buttocks, with the tape parallel to the floor, and the waist-to-hip ratio (WHR) was calculated.

The canonical Adults Treatment Panel III was chosen to define the metabolic syndrome, considering at least three criteria: plasma glucose concentrations ≥100 mg/dL, WC >102/88 cm (male/female), serum HDL concentration <50 mg/dL for women and <40 mg/dL for men, blood pressure ≥130/85 mm Hg, and serum triglyceride concentration ≥150 mg/dL. Insulin resistance was studied by the HOmeostatic Metabolic Assessment (HOMA) method with the formula: fasting insulin (μU/mL) × fasting glucose (mg/dL)/405 (26). A value of HOMA >2 was introduced as limit of the presence of insulin resistance (25). More than five determinations of HOMA in different situations were taken into account. Triglyceride values of subjects who had fasted at least 12/14 h before the blood draw were evaluated, averaging the results of at least two determinations, made on different days. Moreover, the triglyceride/HDL cholesterol ratio was evaluated, considering abnormal values ≥1.65 (men) and ≥1.32 (women) (27, 28). Low-density lipoprotein (LDL) was calculated by the following formula: LDL = total cholesterol − HDL − (triglycerides/5). A new index evaluating hepatic insulin resistance, generated through stepwise linear regression, i.e., 2.2607 + 0.427 × log HOMA was computed (29). The AIP was calculated according to the following formula: AIP = log triglycerides/HDL (30).

### Ultrasonography Features

The spleen diameter, assessed on longitudinal scan, was chosen to evaluate the spleen volume and was carried out by the postero-lateral approach. The classification of hepatic steatosis/NAFLD was based on the following scale of increased echogenity: grade 0 = absent, 1 = light, 2 = moderate, 3 = severe, unraveling the difference between the densities of the liver and the right kidney obtained on the same longitudinal sonographic plane. The levels of brightness of the liver and right kidney were calculated three times directly from the frozen images. Subcutaneous adipose tissue (SAT) and visceral adipose tissue (VAT) were assessed by transverse scanning. SAT was defined as the thickness between the skin-fat interface and the linea alba, avoiding compression. VAT was defined as the distance between the anterior wall of the aorta and the internal face of the recto-abdominal muscle perpendicular to the aorta, measured 1 cm above the umbilicus. When the aortic walls were obscured by bowel gas, a Doppler scan was used to detect them (25). The common carotid artery, the carotid bulb, and the near and far wall segments of the internal carotid artery were scanned bilaterally. Images were obtained in longitudinal sections with a single lateral angle of insonation, optimizing the image for the far wall. IMT was defined as the distance between the ultrasound interfaces of the lumen-intima and media-adventitia. Six manual measurements were performed, with automatic border detection, at equal distances along 1 cm on the far wall of the common carotid, according to the consensus statement from the American Society of Echocardiography Carotid Intima-Media Thickness Task Force, endorsed by the Society for Vascular Medicine (31).

### Blood Pressure Measurements

Systolic/diastolic blood pressure values were obtained averaging three consecutive measurements taken during usual practice hours, after subjects had rested for 5 min in the sitting position.

### Laboratory Data

Blood samples were collected from all participants in the early morning after an overnight fast. An automated hematologic analyzer (Coulter LH750) was used to measure total and differential blood parameters/counts. Testing for serum triglycerides, HDL, fasting insulin, alanine transferase (ALT), gamma-glutamyl transferase, alkaline phosphate, cholinesterase, fasting glucose, fasting insulin, fibrinogen, and ferritin were performed at enrollment by in-house standard procedures. Hs-CRP values, determined by the ELISA test, with reference values between 0.3 and 8.6 mg/L in healthy men and between 0.2 and 9.1 mg/L in healthy women (BioCheck, Inc., CA, USA) were determined in collected and aliquoted samples and frozen at −20°C.

Smoking status was categorized into five classes, i.e., class 1 as non-smokers, class 2 as past smokers, class 3 as social/weekend smokers, class 4 as light/moderate smokers, and finally class 5 as active smokers.

### Statistics

The Kolmogorov–Smirnov test was evaluated to detect whether the variables were normally distributed and accordingly were reported as mean plus SD. Variables not normally distributed were expressed as median (25–75 IQR). The difference in medians was assessed by the Mann–Whitney test for independent samples. Chi-square evaluated frequencies. The two-way cross-tabulation was performed to assess the relation between the severity of obesity and gender on the one hand, and hepatic steatosis grade at ultrasonography on the other. As exploratory step, the degree of the association between single parameters was studied using Spearman’s coefficient of rank correlation (ρ) for non-uniform intervals. To assess the independent effect of a quantitative variable (predictor or explanatory) on eventual prediction of another one, the linear regression analysis (least squares) was used evaluating the coefficient and the *t* (*t*-value). As multivariate analysis, the multiple regression was adopted (Backward Stepwise Selection), first entering all independent variables if *P* = 0.05 into the model (at univariate analysis), and then removing if *P* = 0.1 the non-significant variables sequentially, with a maximum number of 10 steps. IMT was chosen as dependent variable. To appreciate which variables contribute to any extent to the regression equation, the magnitude of standardized coefficient beta (β) was calculated. To assess multicollinearity, variance inflation factor and tolerance were set at >10 and <0.1, respectively. The effect size was calculated evaluating the *R*^2^ (<0.25: small, from 0.25 to 0.64: medium, ≥0.65: large). The maximum number of variables put in the multiple regression analysis never exceeded the ratio of one variable to 10 patients. When analyzing a dataset in which there was one independent variable that determined an outcome, measured with a dichotomous variable—i.e., no smoking status—the logistic regression was performed (OR and 95% CI or coefficient plus SE).

The concordance correlation coefficient (ρ_c_), which measures precision and accuracy, was adopted to evaluate the degree of pair observations at ultrasonography. The statistical analysis was performed operating on Systat 13 (Richmond, CA, USA) and MedCalc Version 15.2.1 ^®^ (Frank Schoonjans) software packages.

## Results

Characteristics of the whole population are shown in Table 1. The most part of patients (89%) showed obesity of second and third class, without any difference for gender (cross-tabulation *P* = 0.4). Specifically, referring to class I/II/II/of obesity there were 7/26/35 females, respectively, while 4/18/20 were males belonging to the aforementioned classes. Concerning the metabolic syndrome presence, 31 females out of 68 and 26 males out of 32 fulfilled the criteria, chi-square 11.29, *P* = 0.0007. Hepatic steatosis was related to the degree of obesity (cross-tabulation *P* = 0.014). Hypertension occurred only in 4 obese patients, while type 2 diabetes mellitus in 16, confirming the low prevalence of co-morbidities. Nearly one-third of patients presented an increase in carotid IMT. Interestingly, only 45 subjects were labeled as active smokers versus 35 self-declared no-smokers or past smokers, while the remainders were light or weekend smokers. Insulin resistance, evaluated as HOMA >2 and present in 56 obese patients, was significantly higher in those diagnosed at US with moderate compared to mild grade of hepatic steatosis (median 2.65 versus 1.42, *P* = 0.006 and Mann–Whitney *U*). Hepatic insulin resistance in males was lower than systemic insulin resistance, expressed as HOMA, i.e., median 2.48 versus 3.49, *P* = 0.003; in females, there was no difference, i.e., median 2.39 versus 2.02, *P* = 0.45, Mann–Whitney *U*. The hematocrit mean value was in both gender generally at the upper limit of normality.

**Table 1:** Characteristics of obese patients with non-alcoholic fatty liver disease (*n* = 100), including metabolic parameters.

Gender M/F	32/68	Age	43.5 (32–52)
BMI	40.7 (37.1–45.1)	BM I > 45 (*n*)	26
WC females (cm)	118.8 ± 16.1	WC males (cm)	132.8 ± 110.6
WHR males	1.02 ± 0.05	WHR females	0.90 ± 0.06
SAT (cm)	2.8 ± 0.06	VAT (cm)	7.3 ± 0.06 2.1
Ferritin females (ng/mL)	38.5 (19.5–70.5)	Ferritin males (ng/mL)	169.7 (140–197)
Fibrinogen (g/L)	323 (285.5–356)	CRP (mg/mL)	0.46 (0.24–0.89)
γ-GT (U/L)	22.5 (14.5–36)	ALT (U/L)	23 (18–33)
Triglycerides (mg/dL)	104.5 (76.5–160.5)	LDL (mg/dL)	169.7 (140.3–1.97)
HDL females (mg/dL)	50.8 ± 11.05	HDL males (mg/mL)	43.7 ± 9.06
Triglyceride/HDL ratio (females)	1.93 (1.38–2.81)	Triglyceride/HDL ratio (males)	3.26 (1.61–4.82)
AIP (females)	0.29 ± 0.23	AIP (males)	0.50 ± 0.36
Hepatic IR	2.4 (2.3–2.5)	HOMA	2.4 (1.32–3.72)
SBP (mm Hg)	130 (120–130)	DBP (mm Hg)	80 (75–100)
IMT > 0.9 mm (yes/no)	35/65	IMT (mm)	0.08 (0.07–0.10)
Hematocrit (females)	39.8 ± 2.99	Hematocrit (males)	44.5 (42.4–46)
White blood cells (mm^3^)	7,750 (6,700–8,850)	Neutrophil lymphocyte ratio	2.06 (1.62–2.31)
Hemoglobin (females) (g/dL)	13.5 ± 1.14	Hemoglobin (males) (g/dL)	15.1 (14.5–15.6)
MCV	86.7 (82.7–90.1)	Platelet count (10^9^/L)	254 ± 54
Spleen longitudinal diameter (mm)	11.23 ± 0.14	Spleen longitudinal diameter > 12 mm (*n*)	15

*Values were expressed as m ± SD or median (25–75 IQR)*.

*n, number of patients; WC, waist circumference; BMI, body mass index; WHR, waist-to-hip ratio; SAT, subcutaneous adipose tissue; VAT, visceral adipose tissue; HDL, high-density lipoprotein cholesterol; CRP, C-reactive protein; ALT, aminotransferase; γ-GT, gamma-glutamyl transglutaminase; LDL, low-density lipoprotein cholesterol; HOMA, HOmeostatic Metabolic Assessment; SBP, systolic blood pressure; DBP, diastolic blood pressure; IMT, intima-media thickness; AIP, atherogenic index of plasma; MCV, mean corpuscular volume*.

### Associations

The most relevant correlations are shown in Table 2.

**Table 2:** The most relevant associations.

IMT	Hematocrit	ρ = 0.353	*P* = 0.003
IMT	Hemoglobin	ρ = 0.28	*P* = 0.004
IMT	Platelets count	ρ = 0.30	*P* = 0.0
Hemoglobin	Mean corpuscular volume	ρ = 0.4	*P* < 0.001
Hematocrit	Hepatic steatosis severity	ρ = 0.23	*P* = 0.021
Hepatic insulin resistance	Hepatic steatosis severity	ρ = 0.27	*P* = 0.007
Triglyceride/HDL ratio	WC	ρ = 0.325	*P* = 0.007
γ-GT	HOMA	ρ = 0.50	*P* = 0.0001
γ-GT	Ferritin	ρ = 0.48	*P* = 0.001
HOMA	Hematocrit	ρ = 0.29	*P* = 0.029

*IMT, intima-media thickness; HOMA, HOmeostatic Metabolic Assessment; γ-GT, gamma-glutamyl transglutaminase; WC, waist circumference; HDL, high-density lipoprotein cholesterol; ALT, alanine transferase*.

*Metabolic syndrome occurred much more frequently in those patients who presented an elevation of both ALT and γ-GT serum levels. (Spearman ρ 0.59 with *P* = 0.001 and 0.41 with *P* = 0.001, respectively)*.

Furthermore, IMT was not related to white blood cells (ρ = 0.09, *P* = 0.35) or neutrophil count (ρ = 0.12, *P* = 0.24). There was a trend of significance concerning the relationship between BMI and platelets count (ρ = 0.19, *P* = 0.056) and between hepatic steatosis grade at US and IMT (ρ = 0.18, *P* = 0.07).

### Univariate Analysis

The most relevant predictions at linear regression are shown in Table 3.

**Table 3:** Predictions at univariate analysis.

		Coefficient	*t*	*P*
IMT	Age	0.0012	6.77	<0.0001
IMT	Hematocrit	0.0026	3.45	0.001
IMT	Platelets count	−0.00000017	−3.09	0.0025
IMT	Mean corpuscular volume	0.0011	3.28	0.0014
HOMA	Hematocrit	0.0045	2.7	0.007

*IMT, intima-media thickness; HOMA, HOmeostatic Metabolic Assessment*.

Intima-media thickness was not predicted by white blood cells count, neutrophils, and neutrophil/lymphocyte ratio, *P* = 0.13, 0.19, and 0.94, respectively. Noticeably, hematocrit was not predicted by age, *P* = 0.17. As collateral finding, MCV was not predicted by WC, *P* = 0.8.

As expected, IMT was predicted by smoking status, coefficient 0.0067, *t* = 3.51, *P* = 0.001. In details, non-smoking status was predicted by hematocrit, OR 0.77, 95% CI = 0.67–0.89, *P* = 0.0002.

### Multivariate Analysis

First of all, studying classical risk factors for atherosclerosis, IMT was predicted only by LDL among the metabolic parameters, i.e., hepatic insulin resistance, HOMA, LDL, HDL, AIP, triglyceride/HDL, with coefficient 0.0001, β = 0.25, *t* = 2.58, *P* = 0.011, *R*^2^ = 0.06.

Adding to the model predicting IMT, (i) blood count factors (hematocrit, MCV, and platelets), (ii) age, and (iii) smoking habit, resulted to be predicted by age and hematocrit, coefficient 0.001, β = 0.55, *t* = 6.57, *P* < 0.001, and coefficient 0.0020, β = 0.30, *t* = 3.199, *P* = 0.0019, *R*^2^ = 0.38, respectively. It is worth underlining that hemoglobin was excluded from the analysis due to collinearity with MCV.

Successively, evaluating the role of anthropometric features, among BMI, WC, WHR, SAT, and VAT, IMT was predicted by VAT alone, with coefficient −0.0085, β = 0.49, *t* = 5.5, *P* < 0.0001, *R*^2^ = 0.24.

Furthermore, age and hematocrit, adjusted for VAT, maintained their predictive value, although there was a reduction of more than 20% of the coefficient, lending credence to the fact that visceral adiposity is a real confounding factor. The results for age were coefficient 0.001, β = 0.47, *t* = 5.3, *P* < 0.0001; for hematocrit: coefficient 0.001, β = 0.24, *t* = 2.48, *P* = 0.014, for VAT: coefficient 0.002, β = 0.225, *t* = 2.2, *P* = 0.025, *R*^2^ = 0.41. Finally, when these three variables predicting IMT, i.e., age, hematocrit, and VAT were adjusted for gender, this variable was excluded from the model, evidencing its lack of influence.

### Reliability

To substantiate the clinical significance of US features, the inter-intra-observational reproducibility of the sonographic estimations was high, with a ρ_c_ of 0.93 and 0.89, respectively.

## Discussion

In our studied population (obese patients of second and third grade, with low prevalence of co-morbidities but suffering from NAFLD), both age and hematocrit values predicted IMT, also after adjusting for other evidenced predictors of IMT, i.e., amount of the visceral adiposity, expressed as VAT and evaluated by US and smoking habit. Noteworthy, the latter was also predicted by hematocrit in line with a previous study that found an increase in hematocrit values among smokers and in heavy smokers, these changes likely predisposing to greater risk of developing atherosclerotic plaques and coronary heart disease (32).

Concerning other blood count components, white blood cells and neutrophil count did not show any relation to IMT in our population. Considering both these blood components as inflammatory markers, interestingly our data on CRP are in agreement with the latter finding, in the sense that this acute phase protein was not associated with IMT in our population, partially in accordance with other results showing that CRP and carotid IMT levels appear to be directly related in women, but not in men in a population of 2,640 subjects, gender equally represented, differently from our study (33).

Analyzing the possible mechanisms underlying the association between hematocrit levels and early atherosclerosis, blood viscosity is central to explaining this phenomenon. Unfortunately, recent literature offers divergent data. In fact, Carallo et al. demonstrate that blood viscosity seems independent of classical coronary heart disease risk factors and is unrelated to hematocrit and plasma viscosity, suggesting a possible direct effect of aging on red blood cells (34). By contrast, the passage time, another index of blood rheology, correlated better with hematocrit (*r* = 0.422) than white blood cell count (*r* = 0.295) and platelet count (*r* = 0.204) (35).

Confronting the aforementioned data, we hypothesize that red blood cells increase the adhesiveness of platelets by the erythrocyte-derived ADP available for platelet activation and causes the dispersion of platelets toward the sub-endothelial surface due to blood flow (36, 37). Experimental studies have suggested that red blood cells promote thrombin generation and that the thrombin concentration is proportionate to hematocrit levels (38, 39). As a result, hematocrit could promote thrombosis and atherosclerosis, and consequently CV disease. The finding that in humans blood viscosity, evaluated as hematocrit, is involved in the endothelial response to an increase in shear stress confirms the role of hematocrit in hypertension and consequently in the atherosclerotic process (40).

The relationship between obesity-related NAFLD and atherosclerosis *via* thrombotic mechanisms should not be overlooked. In fact, in a rat model of NAFLD, induced by feeding a fat-rich diet for 24 weeks, the imbalance of plasma PGI2 and thromboxane A2 (TXA2) levels played a role in the pathogenesis of NAFLD (41). TXA2, a marker of platelet activation, evaluated as serum levels thromboxane B2, is higher in obese subjects than in lean ones, and this might be a clue to their increased CV risk (42).

To draw further conclusions from our results, it is worth mentioning that hematocrit was positively associated with insulin resistance—evaluated as HOMA—an association considered an epiphenomenon of visceral adiposity (18). The latter finding confirms that an interplay between alterations of adipose tissue distribution/function and hematocrit could be involved determining broad effects on in the induction/maintenance of atherosclerosis. As partially supporting evidence, taking into account that insulin resistance is a major underlying mechanism responsible for the “metabolic syndrome,” which is also known as insulin resistance syndrome, authors found that beyond elevated levels of hemoglobin and red blood cells counts hematocrit was significantly associated with clustered components of metabolic syndrome in women (43). It is necessary to highlight that differences in definitions of CV disease risk, as well as ethnic and racial differences may account for the absence of consistency across studies.

We did not assess the prevalence of abnormal blood data in our obese patients, extensively reported elsewhere (44), nor were able to confirm previous data from others concerning the link between hepatic steatosis severity and white blood cells count. Indeed, recent research clearly showed that white blood cells count was a significant factor associated with incident NAFLD in Han Chinese without NAFLD at base line, with a HR of 1.152 in the highest quartile, obtained by Cox proportional hazards regression analysis (45).

The role of NAFLD in predicting IMT was not definitely evident in this study, but we emphasize that the NAFLD severity was not assessed by histology, due to the lack of consent to invasiveness by our obese patients. Supportively, we stress that IMT was predicted by the visceral adiposity, a clear sign of NAFLD presence, directly or indirectly representing a CV risk factor (46).

Accordingly, it should be stressed that visceral adiposity was a real confounding factor in predicting IMT, consequently playing a noticeable role in determining/worsening atherosclerosis. In multiple regression, the lack of retaining of smoking as predictor of IMT is likely due the reduced number of active smokers in our population and multicollinearity with age, according to recent investigation (47).

The apparently surprising negative correlation between IMT and platelets count is likely due to the treatment with anti-platelet drugs and to vitamin B12 and/or folate deficiency, often present in the obese, according to de Ilvar et al. (48).

Coming back to our core finding, when comparing our results with relevant findings from other reports of literature by principal search sources during the last decade, interesting data, although not univocal, was found about the relation between hematocrit levels and CV disease risk factors. In fact, this association has been reported inconsistently. Recent findings suggest that both elevated and decreased hematocrit levels are associated with an increased risk of established CV disease, even though the more significant risk was found in the highest quartile of patients with ischemic stroke and coronary artery disease (49). On the other hand, the study of Arbel et al., found that, in patients with angiographically normal coronary arteries and slow coronary flow, hematocrit levels are associated with slower coronary blood flow (SCF). The phenomenon of SCF in the presence of normal coronary arteries may indicate endothelial dysfunction, which is characteristic of an early stage in the development of atherosclerosis. Despite this association, such patients do not have increased carotid IMT values (50). In line with our results stands a study by Tabara et al. including 1,978 participants from two independent cohorts, which showed that hematocrit was positively associated with insulin resistance and insulin sensitivity. However, this association was lost after further adjustment for visceral fat area and plasma ALT, concluding that this association was epiphenomenon of visceral adiposity and hepatic fat excess (18).

### Limitations

First, we acknowledge that the type of study did not allow us to draw conclusions on the direction of the associations.

Second, ours was a single-center study, and large-scale prospective studies are needed to confirm this association and establish whether this new information is sufficient to modify existing clinical practice.

Possible drawbacks were not having evaluated the adipose districts by the more precise magnetic resonance imaging and NAFLD by histology. Anyway, US-detecting NAFLD is a reliable technique in epidemiological studies (51).

Finally, having selected a population with low rate of co-morbidities, which presented relatively few CV risk factors, has its limitations.

## Conclusion and Future Directions

The combination of hematocrit measurements with one of the “canonical risk factors,” e.g., the patients’ age, can improve our ability to detect an early atherosclerosis in the obese. Future observational studies in this setting, comparing patients with low and high rate of co-morbidities, will play an important role in the decision-making process providing invaluable information on the reliability of hematocrit determinations at the light that these are widely available, easy to carry out and interpret. Specifically, as implications for current practice, hematocrit values can alert physician to the need of making a quick diagnosis of incident cardio-metabolic cases when facing such patients with low rate of co-morbidities but NAFLD. Obese patients affected by various co-morbidities are likely to have more severe or premature atherosclerosis due to combined mechanisms and it would be extremely interesting whether hematocrits still remain a predictor of carotid IMT. Indeed, such patients requiring more frequent physician visits have greater opportunity to undergo screening or to have early symptoms of CV disease investigated.

As corollary of our finding, hematocrit determinations could have a further significant impact on clinical decision, mainly on cutting back the spending of the health care system and consequently costs in a real-world environment. Finally, this study merits further research in the sense that a better and more nuanced understanding of the molecular basis underlying the association between hematocrit and carotid IMT and its pathogenic pathways may lead to new strategies to improve CV health as well as overall health and increase longevity.

## Ethics Statement

This study was carried out in accordance with the recommendations of “the Declaration of Helsinki.” The protocol was approved by the “Federico II University Medical School.” Oral or when possible written informed consent was obtained from all subjects.

## Author Contributions

GT conceived the study. All the authors equally contributed to the analysis and interpretation of data, to the draft of manuscript, shared the content, and accepted that GT submitted the paper on their behalf.

## Conflict of Interest Statement

The authors declare that the research was conducted in the absence of any commercial or financial relationships that could be construed as a potential conflict of interest.
